# Family-Centered Prevention Attenuates the Association Between Structural Racism Risk and Black Adolescents’ Low Self-regulation and Externalizing Behaviors: Secondary Analysis of a Randomized Clinical Trial

**DOI:** 10.1007/s11121-025-01828-5

**Published:** 2025-07-17

**Authors:** Steven M. Kogan, Ava J. Reck, Biplav Tiwari, Janani Rajbhandari Thapha, Sierra Carter, Assaf Oshri, Kalsea Koss, Sun Joo Ahn, Steven Beach, Sycarah Fisher, Emilie Smith, Linhao Zhang

**Affiliations:** 1 College of Family and Consumer Sciences, University of Georgia, Athens, GA USA; 2 College of Public Health, University of Georgia, Athens, GA USA; 3 College of Arts and Sciences, Georgia State University, Atlanta, GA USA; 4 Grady College of Journalism and Mass Communication, University of Georgia, Athens, GA USA; 5 Franklin College of Arts and Sciences, University of Georgia, Athens, GA USA; 6 College of Education, University of Georgia, Athens, GA USA; 7 Inaugural College of Social Science, Michigan State University, East Lansing, MI USA

**Keywords:** Structural racism, Evidence-based prevention, Family-centered intervention, Externalizing problems

## Abstract

Converging evidence underscores the influence of structural racism on the emergence of externalizing behaviors among Black youth. Recent studies suggest that family-centered prevention may attenuate some of the effects of structural racism on youth mental health. Hypotheses were tested regarding the potential of the Strong African American Families (SAAF) preventive intervention (ClinicalTrials.gov Identifier: NCT03590132) to (a) attenuate the influence of structural racism on low self-regulation, (b) attenuate the influence of low self-regulation on externalizing behaviors, and (c) disrupt the pathway from structural racism to externalizing behaviors via low self-regulation. Hypotheses were tested with data from 472 Black youth (mean age = 11.61 at pre-test) and their caregivers participating in a randomized prevention trial. Structural racism was indexed according to a cumulative risk model based on census-level indicators of Black-White disparities in labor force participation, housing, educational attainment, poverty, and a measure of racial segregation. Consistent with hypotheses, we found that SAAF buffered the influence of structural racism risk on low self-regulation. A significant buffering effect was not detected on the path from low self-regulation to externalizing behaviors. Conditional indirect effect analysis suggested that attending SAAF disrupted the pathway linking structural racism to externalizing behaviors via low self-regulation. Study findings underscore the potential benefits of widespread dissemination of family-centered prevention targeting evidence-based protective processes designed for Black youth. Clinical Trial: Registered at Clinicaltrials.gov, NCT03590132, July 5, 2018.

**Supplementary Information:**

The online version contains supplementary material available at 10.1007/s11121-025-01828-5.

## Introduction

Conduct problems disproportionately affect Black^1^ adolescents’ development compared to their peers from other racial/ethnic groups (Danielson et al., 2021). Conduct problems are characterized by actions that violate societal norms or rules; they range from moderate instances of aggression and defiance to conduct disorders and delinquency. There is mixed evidence regarding whether or not Black adolescents engage in more problem behavior than their peers (Kim & Hong, 2024). Compared to their White peers, however, Black adolescents’ behavior is more likely to be interpreted as disruptive or dangerous (Abrams et al., 2021; Perillo et al., 2023), leading to disparities in rates of school suspension and expulsion, referrals to juvenile justice systems, and incarceration in juvenile justice facilities (Burriss et al., 2011; Nowicki, 2018; Puzzanchera et al., 2018). This underscores the importance of investigating routine forms of externalizing behavior among Black youth that may (a) place them at risk for disproportionate reactions or (b) presage future externalizing problems.

Converging evidence underscores the influence of anti-Black racism on the emergence of more severe externalizing-related outcomes (suspensions, incarceration) Black youth experience (Weinberger, 2023). Racism is a system of hierarchical categorization of socially constructed “racial” groups for differential allocation of status, resources, and power in ways that privilege White Americans (Harrell, 2000). It comprises a multilevel, multidimensional, and immersive context, systematically producing racial disparities over the life course and across generations (Gee & Hicken, 2021; Gee et al., 2012). At the individual and interpersonal levels, racism includes personal experiences of unfair treatment based on perceived race as well as exposure to marginalizing messages and stereotypes in everyday life and media contexts. Unlike individual racism, which focuses on personal prejudices and discriminatory actions, structural racism is rooted in the policies, practices, and cultural norms of institutions that advantage one racial group while disadvantaging others (Gee & Hicken, 2021). Although studies have revealed the influence of interpersonal racism on mental health outcomes, in recent years there has been a growing emphasis on the importance of examining the influence of structural racism on adolescent development due to its increasing acknowledgment as a fundamental cause of health inequities (Neblett Jr. & Neal, 2022; Williams & Mohammed, 2013).

Three conceptual pathways inform the influence of racism on externalizing behaviors. First, institutional practices and individual biases lead to authority figures over-interpreting Black youths’ behavior as oppositional or aggressive (Abrams et al., 2021), which can become a self-fulfilling prophecy as youth experience the frustration of stigmatization (Haney-Caron & Fountain, 2020). Second, instances of interpersonal and “everyday” racial discrimination evince consistent links with externalizing symptomology (Benner et al., 2018; Lanier et al., 2017; Smith-Bynum et al., 2014; Vines et al., 2017). Third, racism fosters externalizing behaviors by promoting a proliferation of stressful and traumatic experiences associated with poverty, neighborhood disorganization and crime, underfunded schools, and limited opportunity (Ursache et al., 2022; Williams, 2018). Structural racism increases coping demands, which undermine self-regulatory processes associated with behavioral and emotional self-regulation (Gibbons et al., 2012; Seaton, 2020) which serve as proximal risk factors for externalizing problems.

To date, research on these processes has focused primarily on institutional practices funneling Black youth into the juvenile justice system (Abrams et al., 2021), or linking exposure to individual racial discrimination to externalizing symptomatology (Cave et al., 2020). Linking quantitative indices of structural racism, however, to individual youths’ self-regulatory difficulties and externalizing behaviors is scarce. Moreover, recent studies suggest that family-centered prevention may attenuate some of the effects of racism on youth mental health (Brody et al., 2021; Kogan et al., 2023). For example, Kogan et al. (2023) found that among youth in a trial of the Strong African American Families (SAAF) program, self-reported racial discrimination was linked to increased depression. Comparing prevention and control groups, however, revealed that for youth experiencing SAAF, the negative impact of racial discrimination was attenuated. Other research detected similar effects in family-centered trials (Brody et al., 2021). Although this emerging research sends a powerful message regarding the potential for prevention to attenuate the effects of discrimination, these studies neglect the influence of structural racism. The potential for family-centered prevention to attenuate the effects of structural racism via effects on self-regulatory processes has not been investigated. We address this need in the present study.

**Fig. 1:**
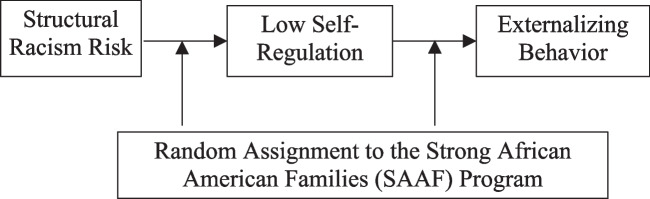
Conceptual model of study hypotheses linking structural racism risk to externalizing behavior

Figure 1 presents study hypotheses. We hypothesize that exposure to structural racism risks will promote low self-regulation among youth, which operates as a proximal risk factor for externalizing behaviors. Family-centered prevention is expected to attenuate the effects of (a) structural racism risk on low self-regulation, (b) the effects of low self-regulation on externalizing behaviors, and (c) disrupt the indirect effect pathway. Empirical and theoretical support for these pathways follows.

Structural racism rests on oppressive practices in and interconnections among societal institutions (i.e., employment, housing, education) that comprise a multilevel context of oppression. Quantifying structural racism quantitatively is a scientific challenge (Neblett Jr. & Neal, 2022). Promising strategies involve place-based indicators of disparities across economic, social, and other domains (e.g., racial segregation, economic disparities, school discipline disparities) (LaFave et al., 2022). Variability in place-based characteristics at varying geographical units (state, county, school district, census tract) is associated with a range of health and other racial disparities (Bailey et al., 2017; Coke & Hayman, 2021; Mesic et al., 2018). Capturing the synergistic effects of structural racism quantitatively, however, is an ongoing challenge (Gee & Hicken, 2021). Structural racism is not simply the average of the disparities observed in a specific place. Institutional practices across domains amplify the marginalizing effects of racism (LaFave et al., 2022). Given the lack of widely accepted measures (Tiwari et al., 2024), and concerns regarding underestimating the influence of structural racism, we adopt a cumulative risk model (Clarke et al., 2014). That is, an individual’s risk for exposure to structural racism may be indexed by the accumulation of place-based risk factors that are theoretically and empirically linked to racial disparities.

We hypothesize that structural racism risk forecasts increases in externalizing behaviors indirectly, by increasing low self-gulation. Low self-regulation is the inability to regulate one’s actions, impulses, or emotions in line with societal norms or personal goals (Billore et al., 2023). It involves difficulties inhibiting inappropriate behaviors, delaying gratification, and considering long-term consequences. Low self-regulation is a robust antecedent of externalizing behaviors (Robson et al., 2020; Wills et al., 2016). Prospective research suggests that individual-level racism undermines the development of self-regulation among Black adolescents (Gibbons et al., 2010, 2012). The multiple barriers to successful life paths that structural racism creates are thought to induce frustration and require high levels of cognitive reserve to cope, thus undermining self-regulation (Adkins-Jackson et al., 2024). Other research links contextual adversities associated with structural racism (poverty, community disorganization, adverse life events) to decrements in self-regulation (Duckworth et al., 2013; Palacios-Barrios & Hanson, 2019). Neighborhood-level inequities may affect self-regulation via multiple pathways, including dysregulation in stress physiology, exposure to toxicants, or limited access to inputs necessary for healthy growth and development (Ursache et al., 2022).

Family-centered prevention is hypothesized to attenuate the pathway from structural racism risk to low self-regulation. Powerful factors affecting the influence of contextual adversity originate in the family environment. Among Black youth, caregivers’ support and nurturance, consistent discipline and monitoring, and positive messages about Black identity can attenuate the influence of adverse environments in general (Beale Spencer et al., 2015; Hardaway et al., 2016) and individual discrimination in particular (Cooper et al., 2013; Dotterer & James, 2018), on youth development. Several recent analyses of prevention trial data suggest that family-centered interventions that target these factors may attenuate the influence of racial discrimination on youth mental health (Berkel et al., 2022; Brody et al., 2021; Kogan & Reck, 2023; Lei et al., 2021). Other studies find family-centered prevention can mitigate some of the effects of community-level risks associated with structural racism risk such as community violence and negative life events (Brody et al., 2019, 2020).

Per Fig. 1, we further hypothesized that assignment to family-centered prevention would attenuate the influence of low self-regulation on externalizing behaviors. Past research finds that for youth with self-regulatory problems, including trait impulsivity and attention deficit disorder symptomology, aspects of the family environment can mitigate the influence of these vulnerabilities on externalizing problems (Duh-Leong et al., 2020; Dvorsky & Langberg, 2016; Lanza and Drabick, 2011a). For example, among children exhibiting impulsive or hyperactive behaviors, those whose families engaged in consistent routines were less likely to have oppositional defiant disorder in adolescence (Lanza and Drabick, 2011b). Structured routines may have increased the predictability of cues in the child’s environment and/or provided additional external controls on behavior that minimized the potential for externalizing problems in adolescence. Other research finds that consistent nurturing behavior is a particularly powerful predictor of reduced misbehavior and externalizing among children high in impulsivity (Stewart et al., 2024). Similarly, parental monitoring reduces risk behavior among youth with impulsive personality traits (Thompson et al., 2015).

We investigated the protective effects of family-centered prevention in the context of a randomized prevention trial of the Strong African American Families (SAAF) program. SAAF is a 7-session, 14-h family skills training program designed for Black American families with an early adolescent youth. The curriculum targets enhancing well-validated protective processes that support adolescent development in the context of challenging environments. These include racial socialization, consistent discipline, parental nurturance and warmth, and a positive Black identity. SAAF has proven efficacious in three trials in enhancing family protective processes, youth self-regulation, and deterring substance use (Brody et al., 2012a; Brody et al., 2004, 2006a, 2006b, 2005; Kogan et al., 2016, 2019). Importantly, recent analyses of trial data suggest that SAAF can attenuate the influence of individual-level racial discrimination on youths’ mental health (Kogan et al., 2023).

### The Present Study

The potential for family-centered prevention in general, and SAAF in particular, to attenuate the influence of structural racism risk on low self-regulation and externalizing behaviors has not been investigated. Per Fig. 1, we tested 3 hypotheses with data from 426 Black families participating in a randomized trial of SAAF. We hypothesized that assignment to SAAF would buffer the effects of structural racism risk on increased low self-regulation. Specifically, we expected that structural racism would have a reduced association with low self-regulation in the SAAF group vs the control group. We further hypothesized that SAAF would buffer the influence of low self-regulation on externalizing behaviors. Finally, we expected that assignment to SAAF would disrupt the racism risk→low self-regulation→externalizing behavior pathways found in the control group. To strengthen inferences regarding structural rather than personal factors explaining our results, we controlled for individual-level racial discrimination. We also enhanced the specificity of our analysis by controlling for poverty status. As we had no expectations that these pathways would be affected by gender, it was controlled as well.

## Methods

Study hypotheses were tested with a secondary analysis of trial data investigating the efficacy of SAAF. Details regarding the trial may be found elsewhere (Kogan et al., 2019) and in Supplement S1. Youth and their primary caregivers (*N* = 472 families) were recruited from eight rural counties in Georgia. School districts provided lists of Black American fifth-grade students whose parents were contacted in random order to discuss participation. Eligibility requirements were the presence in the family of a youth 11 or 12 years of age at pretest who self-identified as African American or Black. Recruitment began on June 7, 2013, and data collection concluded on December 10, 2017. Of the 825 families screened for eligibility, 625 were eligible to participate; 472 were enrolled in the trial (a 76% recruitment rate; Supplement S2).

Youth’s mean age at the baseline assessment was 11.61 years (SD = 0.62). Families had an average of 2.9 children. In 50.8% of these families, the target child was female. The primary caregivers in 88.6% of the families were the youth’s biological mothers, 5.3% were grandmothers, and 3.0% were biological fathers. The caregivers’ mean age was 37.2 years (SD = 8.74). Of the caregivers, 18.2% had less than a high school education, 26.5% had completed high school or obtained a GED, and the remaining 55.3% had at least some college education. A majority of participating families, 64.4%, had family incomes below the federal poverty threshold.

After enrollment and a baseline assessment (Time 1[T1]), participants were assigned randomly to SAAF (*n* = 252) or to a no-treatment control group (*n* = 220). Families completed follow-up assessments at 22 months post-baseline (T2), and 34 months post-baseline (T3). The overall trial design included a second intervention implemented randomly after the T3 follow-up (see S1); we focus here on the effects of SAAF solely, from T1 to T3. Black American field researchers made home visits to collect data using audio computer-assisted self-interviews on laptop computers. Retention from T1 to T3 was 91.7%; retention rates were similar across experimental groups. Informed consent/assent was obtained at all data collection points. Caregivers were paid $100, and youth were paid $40 at each assessment. All study protocols were approved by the university Institutional Review Board (IRB).

### Externalizing Behaviors

At T2 and T3, externalizing behaviors were assessed with five items specifically developed for the assessment of behaviors targeted in the SAAF program. The items were “I sneak out of the house,” “I go places I am not allowed,” “I hang out with kids I am not allowed to,” “I can get out of being punished,” and “I have used cigarettes, alcohol, or marijuana.” The response scale ranged from 1 (*not at all true*) to 4 (*very true*). These externalizing behaviors were operationalized as a latent variable. Because these items lack prior validation evidence, we conducted a confirmatory factor analysis (CFA; see results below). The items evinced theoretically expected associations with low self-regulation (see Table 1), supporting their suitability in assessing externalizing behaviors.

**Table 1:** Correlations, means, and standard deviations of study variables

	1	2	3	4	5	6	7	8	9	10	11	12	13	14	15	16	17
Time 1																	
1. Intervention Status (1 = SAAF)	–																
2. Structural Racism Risk Index	0.07	–															
3. Low Self-Regulation	0.05	0.00	–														
Time 2																	
4. Low Self- Regulation	0.02	0.08	0.55**	–													
5. “… tried alcohol, cigarettes or marijuana”	0.01	− 0.03	0.19**	0.20**	–												
6. “…sneak out of the house”	0.02	− 0.02	0.11*	0.11*	0.24**	–											
7. “ …get out of being punished”	− 0.02	0.01	0.15**	0.14**	0.19**	0.14**	–										
8. “…go places I am NOT allowed to go”	− 0.02	0.02	0.21**	0.16**	0.27**	0.47**	0.26**	–									
9. “… hang out with kids …”	0.03	− 0.03	0.19**	0.24**	0.17**	0.37**	0.23**	0.55**	–								
Time 3																	
10. “…tried alcohol, cigarettes or marijuana”	− 0.01	− 0.03	0.18**	0.22**	0.48**	0.16**	0.10*	0.15**	0.16**	–							
11. “…sneak out of the house”	− 0.01	0.02	0.21**	0.23**	0.28**	0.29**	0.14**	0.22**	0.24**	0.25**	–						
12. “…get out of being punished”	− 0.07	0.09	0.16**	0.17**	0.25**	0.13**	0.32**	0.17**	0.17**	0.20**	0.35**	–					
13. “…go places I am NOT allowed to go”	− 0.09	0.01	0.25**	0.27**	0.13**	0.18**	0.14**	0.19**	0.17**	0.25**	0.43**	0.37**	–				
14. “…hang out with kids …”	− 0.04	0.02	0.24**	0.29**	0.13**	0.24**	0.16**	0.20**	0.28**	0.23**	0.44**	0.37**	0.70**	–			
Covariates																	
15. Poverty (T1)	− 0.01	0.12^*^	0.04	0.07	0.00	− 0.01	0.12*	0.07	0.07	0.03	0.07	0.10	0.07	0.08	–		
16. Sex (1 = female)	− 0.06	− 0.06	0.08	0.05	0.02	0.17**	0.02	0.17**	0.14**	− 0.07	0.09	0.01	0.12*	0.11*	0.03	–	
17. Interpersonal racism (T2)	0.06	0.00	0.25**	0.20**	0.12*	0.21**	0.11*	0.19**	0.16**	0.09	0.19**	0.16**	0.12*	0.14**	0.06	0.10*	–
Means	0.54	1.54	0.00	0.00	0.24	1.17	1.89	1.29	1.42	0.32	1.25	1.88	1.29	1.43	0.69	.51	20.17
Standard deviations	0.50	1.32	1.50	1.50	0.65	0.58	1.02	0.73	0.82	0.69	0.69	0.99	0.74	0.83	0.47	.50	9.20

*p < 0.05 (2-tailed); **p < 0.01 (2-tailed)

### Structural Racism Risk

We linked participants’ census tracts at T1 to place-based indicators of structural racism specified by Mesic et al. (2018). Accurate address information was provided by 458 participants. Missing data due to lack of a valid address was not associated with any study variables. Participants resided in 49 different census tracts at T1. Structural racism indicators included Black-White disparities in poverty, rental housing, labor force participation, household income, and educational attainment and racial segregation. Residential segregation by race was assessed with the dissimilarity index (Bureau, 2021) which is the percentage of a group’s population (in our case, Blacks) that would have to change residence for each neighborhood (in our case, census blocks) to have the same percentage of that group as the metropolitan area (in our case, census tract) overall, i.e.,

$$\frac{\sum_{i=1}^{n}\left({t}_{i}\left|{p}_{i}-P\right|\right)}{2TP\left(1-P\right)}$$

where, *n* = number of census blocks within the census tract, *t*_i_ = total population of census block “i”, *p*_i_ = proportion of the census block’s population that is minority, *P* = proportion of census tract’s population that is minority, and *T* = total population of census tract. The dissimilarity index ranged from 0 to 100, where a higher value indicated higher segregation between races.

Black-White disparities in poverty, rental housing, labor force participation, and educational attainment were the ratio of the proportion of blacks to the proportion of whites living under the poverty level, living in rental housing, not participating in the labor force, and without a college degree, respectively. Whereas the Black-White disparity in household income was the ratio of White median annual household income to black median annual household income. All of these indices were calculated at the census tract level.

Adopting an additive risk model per our discussion in the introduction, we created a structural racism risk index by dichotomizing each factor. This decision was supported by the lack of strong intercorrelations among the indicators (avg *r* = 0.21). Due to the lack of natural cutoffs, we elected to use a conservative approach to strengthen inferences regarding the presence of risk; each factor was dichotomized based on a quartile split (top quartile = the risk was present [1], lower quartiles = risk not present [0]; (Gelman & Park, 2009). We then summed across structural racism risk indicators (range = 0–6).

### Low Self-regulation

Low self-regulation was assessed from youth and caregiver perspectives at T1 and T2. Youth completed the low self-regulation subscale of Wills’ measure (Wills et al., 2007). The 8-item subscale included items such as “I have to have everything right away,” “I often do things without stopping to think,” and “I often get involved in things I later wished I could get out of.” Youth responded on a scale ranging from 1 (*not at all true*) to 4 (*pretty true*). Cronbach’s alphas were 0.80 at T1 and 0.73 at T2. Parents completed the low self-regulation subscale from Humphrey’s measure (Humphrey, 1982). The 6-item subscale included items such as “How often is your child distracted from work or responsibilities?” How often does your child make careless mistakes because he/she rushes?” and “How often does your child have to have things right away?” Cronbach’s alphas were 0.80 at T1 and 0.74 at T2. The scales were significantly associated (T1 r = 0.33, *p* < 0.001, T2 r = 0.30, *p* < 0.001) and thus were averaged to form a multi-reporter indicator of self-regulation at T1 and T2.

### Covariates

Youth identified as male or female (non-binary information was not collected in this study). Parents provided their monthly income from which poverty status was coded at T1. At T2, youth completed the 13-item Racist Hassles Scale ((Brody et al., 2006a, 2006b); alpha = 0.95). Example items include “How often have you been blamed for something or treated suspiciously (as if you have done something or will do something wrong) because of your race?” How often did others respond to you as if they were afraid because of your race? And “How often have you been watched or followed while in public because of your race?” The time frame was the past six months; respondents answered on a scale ranging from 1 (*never*) to 4 (*frequently*).

### Plan of Analysis

Moderated mediation analyses were conducted in the structural equation modeling framework using Mplus (version 8) within a structural equation framework (Muthén & Muthén, 2009). Participant attrition was not associated with study variables, suggesting that the data was suitable for full-information likelihood estimation (Enders, 2001). Model fit was assessed using multiple fit indices. Comparative fit index (CFI) values greater than 0.95, root mean square error of approximation (RMSEA) values less than 0.08, and a chi-square/degrees of freedom (*χ*^2^/*df)* ratio less than 3.0 were used as indicators of good model fit (Hu & Bentler, 1999). Preliminary analyses included a CFA of the latent outcome with a test for invariance across the intervention and control group, and across time longitudinally. Hypotheses were tested with multi-group path analyses comparing the Structural Racism RiskLow Self Self-regulationExternalizing Behavior pathway based on random assignment to SAAF vs Control. Direct and indirect pathways, including the covariates (sex, T2 interpersonal racial discrimination, T1 SES risk), were entered simultaneously. Conditional indirect effects were estimated with 10,000 bootstrapped samples, and significance was determined using a 95% confidence interval. All analyses were clustered based on census tract membership. Model pathways were compared using a log-likelihood ratio test (Satorra, 2000). Autoregressive controls for one year prior were included for low self-regulation and externalizing behaviors.

## Results

Study variables’ means, standard deviations, and associations are presented in Table 1. A unidimensional CFA of the externalizing behavior data fit well at T2: *χ*^2^ (5) = 9.87, *p* < 0.001, RMSEA = 0.05, CFI = 0.95, and at T3: *χ*^2^ (5) = 0.50, RMSEA = 0.05, CFI = 0.98. The factor loadings ranged from 0.32 to 0.83, were significant (*p* < 0.05), and loaded on the latent factor in the expected direction. Examination of metric invariance with multi-group analyses revealed no significant differences in item loadings for the SAAF and control groups at each wave T2: Δ*χ*^2^(4) = 2.50, *p* = 0.64 and T3: Δ*χ*^2^(4) = 4.34, *p* = 0.36. Additionally, no significant differences in item loadings were detected longitudinally across T2 and T3: Δχ^2^(4) = 10.14, *p* = 0.21.

**Fig. 2:**
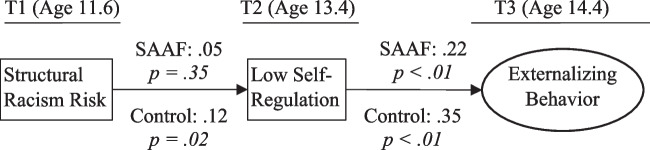
Multigroup analysis of study hypotheses. Note: Standardized estimates presented. T1 sex, poverty, and low self-regulation and T2 externalizing behavior controlled but not pictured

Figure 2 presents the test of study hypotheses. The model fit the data well: χ2 (193) = 342.73, RMSEA = 0.06, and CFI = 0.95. The structural racism index predicted an increase in youth’s low self-regulation one year later for the control group (*β* = 0.12, *p* = 0.02) but not for the intervention group (*β* = 0.05, *p* = 0.35). The link between the structural racism index and low self-regulation was significantly different between the control group and intervention group (−2LLΔ [1] = 6.10, *p* = 0.01). Low self-regulation significantly predicted an increase in externalizing behaviors one year later for both the control group (*β* = 0.35, *p* < 0.01) and the intervention group (*β* = 0.22, *p* < 0.01). The link between low self-regulation and externalizing behaviors was not significantly different between the control group and intervention group (-LLΔ [Δdf] = 2.1[1], *p* = 0.16). Bootstrapped indirect effects revealed that structural racism risk indirectly predicted increases in externalizing behaviors via low self-regulation for the control group (Est. = 0.04, 95% CI = 0.02, 0.06) but not for the intervention group (Est. = 0.01, 95% CI =  − 0.01, 0.04).

## Discussion

Racism is a multilevel, immersive system of oppression that undermines Black Americans’ wellbeing and affects the prevalence and consequences of externalizing behaviors. This context underscores two interrelated realities. First, there is an urgent need to dismantle oppressive social structures through policy, institutional action, and advocacy to promote equity, inclusion, and social justice. Given the intransigence of anti-Black racism in US history, however, there is also an urgent need to develop, refine, and disseminate behavioral interventions that can promote Black individuals’ ability to survive, succeed, and thrive despite racism.

Extending past research that found family-centered prevention may attenuate the effects of individual-level racial discrimination on Black youths’ mental health, we investigated if assignment to the SAAF program attenuated the influence of structural racism risk on low self-regulation and of low self-regulation on misbehavior. Our analysis controlled for individual-level discrimination to better support inferences specifically regarding structural influences. Consistent with hypotheses, we found that for youth in the control group, structural racism risk promoted problems with self-regulation; conversely, this link did not manifest for youth assigned to SAAF. The difference between experimental groups in the prospective association of structural racism risk and low self-regulation was statistically significant, suggesting a buffering effect of the structural racism➜low self-regulation association. Conditional indirect effect analysis revealed a significant indirect effect linking structural racism to externalizing behaviors via low self-regulation for the control group, but not for the SAAF group.

The study findings are consistent with prior empirical research, which shows that Black youth benefitted from a significant buffering influence due to their participation in family-centered prevention. These effects have been detected in the context of exposure to individual-level racial discrimination (Berkel et al., 2022; Brody et al., 2021; Kogan et al., 2023; Lei et al., 2021) and neighborhood-level risk factors (Brody et al., 2019, 2020). We extend these findings specifically to the potential influence of structural racism risk on low self-regulation and externalizing behaviors. Together, these findings are noteworthy given their investigation in five different randomized trials with four different intervention programs. All are relatively brief (5–7 sessions, 10–14 h) skills-based programs. These programs address early and middle adolescent youth, targeting a range of protective factors associated with healthy adolescent development among Black youth including parental racial socialization, effective parenting and co-parenting practices, and positive Black identity. Notably, these programs represent culturally and ecologically tailored interventions rather than generic or surface adaptations of interventions for Black youth. This accumulating body of studies underscores the potential of widespread dissemination of family-centered prevention designed specifically for Black youth.

Study findings are consistent with decades of research across diverse social science disciplines underscoring the power of family relationships and parenting practices to shield children and youth from the influence of contextual adversity on development (Lynch et al., 2021). Despite increasing individuation in adolescence, family dynamics and parenting processes profoundly shape adolescents’ development and response to contextual adversity. Although racism is deeply embedded within US society (Breland & Stanton, 2022; Buggs et al., 2020), Black families are not passive recipients of social inequities. They actively challenge the demands of racism in their lives, which includes the development of responses to navigate racism in ways that support youth development (Wint et al., 2022) and contest the assumptions of racist hierarchies (Anderson, 2019; Payne & Brown, 2010). Study findings support the design, implementation, and dissemination of prevention strategies that target evidence-based protective processes such as racial socialization, effective parenting practices, and racial identity. An important caveat must be noted. A sole focus on augmenting protective processes among minoritized youth risks a subtle form of racism and stigmatization where lay audiences may suggest that the “answer” to racism is “empowering” those who must cope with systemic inequity. We contend that empowerment must be only one strand of a larger policy mandate that targets structural and institutional racism.

Contrary to study hypotheses, we did not find that SAAF moderated the influence of low self-regulation on internalizing. Although the strength of the association was reduced in the SAAF group, the comparison of coefficients was not statistically significant. The direction of the effects, however, is promising, and investigation with larger samples is warranted in future research. The potential for this kind of effect bolsters the potential utility of family-centered prevention to operate in indicated prevention contexts with youth who already evince vulnerabilities. Investigation of buffering effects in general, and those related to social inequities in particular, provides a novel method for evaluating the value of an intervention, extending how effectiveness is understood (Brody et al., 2012b). In the context of multi-component interventions with multiple intervention-targeted mediators, direct effect analyses may be limited. As multiple pathways and risk factors are associated with misbehavior, externalizing problems, and other outcomes, interventions may (a) benefit children in different ways and/or (b) achieve benefits via different mediating pathways. From this perspective, prevention programs may be considered a set of resilience resources rather than a singular intervention designed to change a specific risk factor and outcome. It may be the case that the question of under what contextual circumstances an intervention was helpful is as informative as finding a direct effect.

### Strengths and Limitations

We assessed structural racism using a cumulative, place-based risk model. To date, studies that operationalize structural racism risk using place-based data and linking this variability to individual-level behavior (rather than area rates of disease or crime) are scarce. Integrating a place-based metric of structural racism risk in the context of prevention trial data is novel. We acknowledge, however, that this is a rapidly developing area and that novel metrics and new indicators are being examined. Future research is needed that considers alternative models of structural racism, including consideration of sub-factors and profiles and interactions between indicators.

We developed a latent variable for externalizing behaviors with items derived from a project-developed scale. Future research would benefit from the use of standardized measures and multiple reporters. Although we used items developed to assess intervention targets linked to externalizing risk, there are benefits to this approach in the context of a prevention study. Rather than assessing potential psychopathology, we focused on routine and common behavior that, while externalizing in nature, does not necessarily suggest disorder. This allows us to better consider how structural racism may influence youth behavior in a community sample where high rates of serious externalizing problems would not be expected. This is particularly important with young Black adolescents for whom the impact of externalizing behaviors may be amplified by structural racism. Notably, our latent construct fit the data well, was invariant longitudinally and across experimental groups, and the items evinced theoretically expected associations with low self-regulation. Additional research is also needed that considers a range of potential mechanisms through which family-centered intervention attenuates the influence of structural racism. Finally, the sample was composed of rural families in Georgia; findings may not generalize to other geographic regions.

## Supplementary Information

Below is the link to the electronic supplementary material.

Supplementary file1 (DOCX 113 KB)

Supplementary file2 (DOCX 31 KB)

## Footnotes

1 “Black” refers to individuals of African descent. Race is a socially constructed category. Through racism, race has biological, psychological, and sociological consequences.

## References

[CR1] Abrams, L. S., Mizel, M. L., & Barnert, E. S. (2021). The criminalization of young children and overrepresentation of black youth in the juvenile justice system. *Race and Social Problems,**13*, 73–84.

[CR2] Adkins-Jackson, P. B., Kim, B., Higgins Tejera, C., Ford, T. N., Gobaud, A. N., Sherman-Wilkins, K. J., Turney, I. C., Avila-Rieger, J. F., Sims, K. D., Okoye, S. M., Belsky, D. W., Hill-Jarrett, T. G., Samuel, L., Solomon, G., Cleeve, J. H., Gee, G., Thorpe, R. J., Jr., Crews, D. C., Hardeman, R. R., . . . Manly, J. J. (2024). “Hang ups, let downs, bad breaks, setbacks”: Impact of structural socioeconomic racism and resilience on cognitive change over time for persons racialized as Black. *Health Equity*,* 8*(1), 254–268. 10.1089/heq.2023.0151 10.1089/heq.2023.0151 PMC11043623 38665381

[CR3] Anderson, L. A. (2019). Rethinking resilience theory in African American families: Fostering positive adaptations and transformative social justice. *Journal of Family Theory & Review,**11*(3), 385–397. 10.1111/jftr.12343

[CR4] Bailey, Z. D., Krieger, N., Agénor, M., Graves, J., Linos, N., & Bassett, M. T. (2017). Structural racism and health inequities in the USA: Evidence and interventions. *The Lancet,**389*(10077), 1453–1463. 10.1016/S0140-6736(17)30569-X 28402827

[CR5] Beale Spencer, M., Harpalani, V., Cassidy, E., Jacobs, C. Y., Donde, S., Goss, T. N., Muñoz‐Miller, M., Charles, N., & Wilson, S. (2015). Understanding vulnerability and resilience from a normative developmental perspective: Implications for racially and ethnically diverse youth. *Developmental psychopathology: Volume one: Theory and method*, 627–672.

[CR6] Benner, A. D., Wang, Y., Shen, Y., Boyle, A. E., Polk, R., & Cheng, Y.-P. (2018). Racial/ethnic discrimination and well-being during adolescence: A meta-analytic review. *American Psychologist,**73*(7), 855. 30024216 10.1037/amp0000204 PMC6172152

[CR7] Berkel, C., Murry, V. M., Thomas, N. A., Bekele, B., Debreaux, M. L., Gonzalez, C., & Hanebutt, R. A. (2022). The strong African American families program: Disrupting the negative consequences of racial discrimination through culturally tailored, family-based prevention. *Prevention Science*. 10.1007/s11121-022-01432-x 36107276 10.1007/s11121-022-01432-x PMC11178634

[CR8] Billore, S., Anisimova, T., & Vrontis, D. (2023). Self-regulation and goal-directed behavior: A systematic literature review, public policy recommendations, and research agenda. *Journal of Business Research,**156*, 113435. 10.1016/j.jbusres.2022.113435

[CR9] Breland, J. Y., & Stanton, M. V. (2022). Anti-Black racism and behavioral medicine: Confronting the past to envision the future. *Translational Behavioral Medicine,**12*(1), ibab090. 34244794 10.1093/tbm/ibab090

[CR10] Brody, G. H., Murry, V. M., Gerrard, M., Gibbons, F. X., Molgaard, V., McNair, L., Brown, A. C., Wills, T. A., Spoth, R. L., Luo, Z., Chen, Y., & Neubaum-Carlan, E. (2004). The strong African American families program: Translating research into prevention programming. *Child Development,**75*, 900–917. 15144493 10.1111/j.1467-8624.2004.00713.x

[CR11] Brody, G. H., Murry, V. M., McNair, L., Chen, Y.-F., Gibbons, F. X., Gerrard, M., & Wills, T. A. (2005). Linking changes in parenting to parent-child relationship quality and youth self-control: The strong African American families program [Empirical Study Quantitative Study]. *Journal of Research on Adolescence,**15*(1), 47–69. 10.1111/j.1532-7795.2005.00086.x

[CR12] Brody, G. H., Chen, Y. F., Murry, V. M., Ge, X., Simons, R. L., Gibbons, F. X., Gerrard, M., & Cutrona, C. E. (2006a). Perceived discrimination and the adjustment of African American youths: A five-year longitudinal analysis with contextual moderation effects. *Child Development,**77*(5), 1170–1189. 16999791 10.1111/j.1467-8624.2006.00927.x

[CR13] Brody, G. H., Murry, V. M., Kogan, S. M., Gerrard, M., Gibbons, F. X., Molgaard, V., Brown, A. C., Anderson, T., Chen, Y.-F., Luo, Z., & Wills, T. A. (2006b). The strong African American families program: A cluster-randomized prevention trial of long-term effects and a mediational model. *Journal of Consulting and Clinical Psychology,**74*(2), 356–366. 10.1037/0022-006x.74.2.356 16649880 10.1037/0022-006X.74.2.356

[CR14] Brody, G. H., Kogan, S. M., & Grange, C. M. (2012a). Translating longitudinal, developmental research with rural African American families into prevention programs for rural African American youth. In V. Maholmes & R. B. King (Eds.), *Oxford Handbook of Poverty and Child Development* Oxford University Press.

[CR15] Brody, G. H., Yu, T., Chen, Y., Kogan, S. M., & Smith, K. E. (2012b). The adults in the making program: Long-term protective stabilizing effects on alcohol use and substance use problems for rural African American emerging adults. *Journal of Consulting and Clinical Psychology,**80*(1), 17–28. 22182263 10.1037/a0026592 PMC3265673

[CR16] Brody, G. H., Yu, T., Miller, G. E., Ehrlich, K. B., & Chen, E. (2019). Preventive parenting intervention during childhood and young black adults’ unhealthful behaviors: A randomized controlled trial. *Journal of Child Psychology and Psychiatry, and Allied Disciplines,**60*(1), 63–71. 10.1111/jcpp.12968 30203840 10.1111/jcpp.12968 PMC10589912

[CR17] Brody, G. H., Yu, T., Miller, G. E., & Chen, E. (2020). A family-centered prevention ameliorates the associations of low self-control during childhood with employment income and poverty status in young African American adults. *Journal of Child Psychology and Psychiatry, and Allied Disciplines,**61*(4), 425–435. 10.1111/jcpp.13139 31657021 10.1111/jcpp.13139 PMC7078058

[CR18] Brody, G. H., Yu, T., Chen, E., Miller, G. E., Barton, A. W., & Kogan, S. M. (2021). Family-centered prevention effects on the association between racial discrimination and mental health in Black adolescents: Secondary analysis of 2 randomized clinical trials. *JAMA Network Open,**4*(3), e211964–e211964. 10.1001/jamanetworkopen.2021.1964 33760092 10.1001/jamanetworkopen.2021.1964 PMC7991970

[CR19] Buggs, S. G., Pittman Claytor, C., García, S. J., Imoagene, O., Keith, V., Khoshneviss, H., Lee, C., Mayorga-Gallo, S., Ray, V. E., & Roth, W. D. (2020). Systemic anti-black racism must be dismantled: Statement by the American Sociological Association section on racial and ethnic minorities. In (Vol. 6, pp. 289–291): SAGE Publications Sage, CA.

[CR20] Bureau, U. S. C. (2021). *Housing patterns: Appendix B: Measures of residential segregation*. US Census Bureau. Retrieved 7/2/24 from https://www.census.gov/topics/housing/housing-patterns/guidance/appendix-b.html

[CR21] Burriss, F. A., Breland-Noble, A. M., Webster, J. L., & Soto, J. A. (2011). Juvenile mental health courts for adjudicated youth: Role implications for child and adolescent psychiatric mental health nurses. *Journal of Child and Adolescent Psychiatric Nursing,**24*(2), 114–121. 10.1111/j.1744-6171.2011.00276.x 21501288 10.1111/j.1744-6171.2011.00276.x PMC3807871

[CR22] Cave, L., Cooper, M. N., Zubrick, S. R., & Shepherd, C. C. J. (2020). Racial discrimination and child and adolescent health in longitudinal studies: A systematic review. *Social Science & Medicine,**250*, 112864. 10.1016/j.socscimed.2020.112864 32143088 10.1016/j.socscimed.2020.112864

[CR23] Clarke, P., Morenoff, J., Debbink, M., Golberstein, E., Elliott, M. R., & Lantz, P. M. (2014). Cumulative exposure to neighborhood context: Consequences for health transitions over the adult life course. *Research on Aging,**36*(1), 115–142. 10.1177/0164027512470702 24465068 10.1177/0164027512470702 PMC3900407

[CR24] Coke, L. A., & Hayman, L. L. (2021). The impact of structural racism on cardiovascular health. *Journal of Cardiovascular Nursing,**36*(3), 196–197. 33830719 10.1097/JCN.0000000000000807

[CR25] Cooper, S. M., Brown, C., Metzger, I., Clinton, Y., & Guthrie, B. (2013). Racial discrimination and African American adolescents’ adjustment: Gender variation in family and community social support, promotive and protective factors. *Journal of Child and Family Studies,**22*(1), 15–29. 10.1007/s10826-012-9608-y

[CR26] Danielson, M. L., Bitsko, R. H., Holbrook, J. R., Charania, S. N., Claussen, A. H., McKeown, R. E., Cuffe, S. P., Owens, J. S., Evans, S. W., & Kubicek, L. (2021). Community-based prevalence of externalizing and internalizing disorders among school-aged children and adolescents in four geographically dispersed school districts in the United States. *Child Psychiatry & Human Development,**52*, 500–514. 32734339 10.1007/s10578-020-01027-z PMC8016018

[CR27] Dotterer, A. M., & James, A. (2018). Can parenting microprotections buffer against adolescents’ experiences of racial discrimination? *Journal of Youth and Adolescence,**47*(1), 38–50. 10.1007/s10964-017-0773-6 29052120 10.1007/s10964-017-0773-6

[CR28] Duckworth, A. L., Kim, B., & Tsukayama, E. (2013). Life stress impairs self-control in early adolescence. *Frontiers in Psychology,**3*, 608. 23443890 10.3389/fpsyg.2012.00608 PMC3581033

[CR29] Duh-Leong, C., Fuller, A., & Brown, N. M. (2020). Associations between family and community protective factors and attention-deficit/hyperactivity disorder outcomes among US children. *Journal of Developmental & Behavioral Pediatrics,**41*(1), 1–8. 31464826 10.1097/DBP.0000000000000720

[CR30] Dvorsky, M. R., & Langberg, J. M. (2016). A review of factors that promote resilience in youth with ADHD and ADHD symptoms. *Clinical Child and Family Psychology Review,**19*, 368–391. 27747466 10.1007/s10567-016-0216-z

[CR31] Enders, C. K. (2001). The performance of the full information maximum likelihood estimator in multiple regression models with missing data. *Educational and Psychological Measurement,**61*(5), 713–740.

[CR32] Gee, G. C., & Hicken, M. T. (2021). Structural racism: The rules and relations of inequity. *Ethnicity & Disease,**31*(Suppl 1), 293. 34045831 10.18865/ed.31.S1.293 PMC8143846

[CR33] Gee, G. C., Walsemann, K. M., & Brondolo, E. (2012). A life course perspective on how racism may be related to health inequities. *American Journal of Public Health,**102*(5), 967–974. 10.2105/ajph.2012.300666 22420802 10.2105/AJPH.2012.300666 PMC3483932

[CR34] Gelman, A., & Park, D. K. (2009). Splitting a predictor at the upper quarter or third and the lower quarter or third. *The American Statistician,**63*(1), 1–8. 10.1198/tast.2009.0001

[CR35] Gibbons, F. X., O’Hara, R. E., Stock, M. L., Gerrard, M., Weng, C.-Y., & Wills, T. A. (2012). The erosive effects of racism: Reduced self-control mediates the relation between perceived racial discrimination and substance use in African American adolescents. *Journal of Personality and Social Psychology,**102*(5), 1089–1104. 10.1037/a0027404 22390225 10.1037/a0027404 PMC3341491

[CR36] Gibbons, F. X., Etcheverry, P. E., Stock, M. L., Gerrard, M., Weng, C. Y., Kiviniemi, M., & O'Hara, R. E. (2010). Exploring the link between racial discrimination and substance use: What mediates? What buffers? *Journal of personality and social psychology*, *99*(5), 785–801. http://proxy-remote.galib.uga.edu/login?url=http://search.ebscohost.com/login.aspx?direct=true&db=mnh&AN=20677890&site=ehost-live 10.1037/a0019880 PMC3314492 20677890

[CR37] Haney-Caron, E., & Fountain, E. (2020). Young, Black, and wrongfully charged: A cumulative disadvantage framework. *Dickinson l. Rev.,**125*, 653.

[CR38] Hardaway, C. R., Sterrett-Hong, E., Larkby, C. A., & Cornelius, M. D. (2016). Family resources as protective factors for low-income youth exposed to community violence. *Journal of Youth and Adolescence,**45*(7), 1309–1322. 10.1007/s10964-015-0410-1 26748921 10.1007/s10964-015-0410-1

[CR39] Harrell, S. P. (2000). A multidimensional conceptualization of racism-related stress: Implications for the well-being of people of color. *American Journal of Orthopsychiatry,**70*(1), 42–57. 10702849 10.1037/h0087722

[CR40] Hu, L. T., & Bentler, P. M. (1999). Cutoff criteria for fit indexes in covariance structure analysis: Conventional criteria versus new alternatives. *Structural Equation Modeling: A Multidisciplinary Journal,**6*(1), 1–55.

[CR41] Humphrey, L. L. (1982). Children’s and teachers’ perspectives on children’s self-control: The development of two rating scales. *Journal of Consulting and Clinical Psychology,**50*(5), 624–633. 10.1037/0022-006X.50.5.624 7142538 10.1037//0022-006x.50.5.624

[CR42] Kim, C., & Hong, R. (2024). Examining behavioral variations in disadvantaged adolescents: A cross-racial study of African, Latinx, and Asian American adolescents. *Youth & Society*,* 0*(0), 0044118X241240495. 10.1177/0044118x241240495

[CR43] Kogan, S. M., Lei, M.-K., Brody, G. H., Futris, T. G., Sperr, M., & Anderson, T. (2016). Implementing family-centered prevention in rural African American communities: A randomized effectiveness trial of the Strong African American Families Program [journal article]. *Prevention Science,**17*(2), 248–258. 10.1007/s11121-015-0614-3 26459373 10.1007/s11121-015-0614-3 PMC5911919

[CR44] Kogan, S. M., Bae, D., Lei, M. K., & Brody, G. H. (2019). Family-centered alcohol use prevention for African American adolescents: A randomized clinical trial. *Journal of Consulting and Clinical Psychology,**87*(12), 1085–1092. 10.1037/ccp0000448 31613129 10.1037/ccp0000448 PMC6856406

[CR45] Kogan, S. M., Kwon, E., Brody, G. H., Azarmehr, R., Reck, A. J., Anderson, T., & Sperr, M. (2023). Family-centered prevention to reduce discrimination-related depressive symptoms among Black adolescents: Secondary analysis of a randomized clinical trial. *JAMA Network Open,**6*(11), e2340567–e2340567. 10.1001/jamanetworkopen.2023.40567 37910105 10.1001/jamanetworkopen.2023.40567 PMC10620615

[CR46] Kogan, S. M., & Reck, A. (2023). Family-centered prevention, structural racism, and externalizing problems. *unpublished manuscript*, *University of Georgia*.

[CR47] LaFave, S. E., Bandeen-Roche, K., Gee, G., Thorpe, R. J., Li, Q., Crews, D., Samuel, L., Cooke, A., Hladek, M., & Szanton, S. L. (2022). Quantifying older Black Americans’ exposure to structural racial discrimination: How can we measure the water in which we swim? *Journal of Urban Health*. 10.1007/s11524-022-00626-6 35486285 10.1007/s11524-022-00626-6 PMC9561453

[CR48] Lanier, Y., Sommers, M. S., Fletcher, J., Sutton, M. Y., & Roberts, D. D. (2017). Examining racial discrimination frequency, racial discrimination stress, and psychological well-being among Black early adolescents. *Journal of Black Psychology,**43*(3), 219–229.

[CR49] Lanza, H. I., & Drabick, D. A. (2011a). Family routine moderates the relation between child impulsivity and oppositional defiant disorder symptoms. *Journal of Abnormal Child Psychology,**39*(1), 83–94. 20690009 10.1007/s10802-010-9447-5 PMC3066087

[CR50] Lanza, H. I., & Drabick, D. A. (2011b). Family routine moderates the relation between child impulsivity and oppositional defiant disorder symptoms. *Journal of Abnormal Child Psychology,**39*(1), 83–94. 10.1007/s10802-010-9447-5 20690009 10.1007/s10802-010-9447-5 PMC3066087

[CR51] Lei, M. K., Lavner, J. A., Carter, S. E., Hart, A. R., & Beach, S. R. (2021). Protective parenting behavior buffers the impact of racial discrimination on depression among Black youth. *Journal of Family Psychology*. 10.1037/fam0000822 PMC8225556 33705179

[CR52] Lynch, S. J., Sunderland, M., Newton, N. C., & Chapman, C. (2021). A systematic review of transdiagnostic risk and protective factors for general and specific psychopathology in young people. *Clinical Psychology Review,**87*, 102036. 33992846 10.1016/j.cpr.2021.102036

[CR53] Mesic, A., Franklin, L., Cansever, A., Potter, F., Sharma, A., Knopov, A., & Siegel, M. (2018). The relationship between structural racism and black-white disparities in fatal police shootings at the state level. *Journal of the National Medical Association,**110*(2), 106–116. 29580443 10.1016/j.jnma.2017.12.002

[CR54] Muthén, B., & Muthén, B. O. (2009). *Statistical analysis with latent variables* (Vol. 123). Wiley New York.

[CR55] Neblett, E. W., Jr., & Neal, A. J. (2022). Measuring institutional and structural racism in research on adolescence and developmental science. *Journal of Research on Adolescence,**32*(4), 1280–1284. 10.1111/jora.12810 36519420 10.1111/jora.12810 PMC10108306

[CR56] Nowicki, J. M. (2018). K-12 Education: Discipline disparities for Black students, boys, and students with disabilities. Report to Congressional Requesters. GAO-18–258. *US Government Accountability Office*.

[CR57] Palacios-Barrios, E. E., & Hanson, J. L. (2019). Poverty and self-regulation: Connecting psychosocial processes, neurobiology, and the risk for psychopathology. *Comprehensive Psychiatry,**90*, 52–64. 30711814 10.1016/j.comppsych.2018.12.012

[CR58] Payne, Y. A., & Brown, T. M. (2010). The educational experiences of street-life-oriented black boys: How black boys use street life as a site of resilience in high school. *Journal of Contemporary Criminal Justice,**26*(3), 316–338.

[CR59] Perillo, J. T., Sykes, R. B., Bennett, S. A., & Reardon, M. C. (2023). Examining the consequences of dehumanization and adultification in justification of police use of force against Black girls and boys. *Law and Human Behavior,**47*(1), 36. 36931848 10.1037/lhb0000521

[CR60] Puzzanchera, C., Hockenberry, S., Sladky, T., & Kang, W. (2018). Juvenile residential facility census databook. *Pittsburgh, PA: Office of Juvenile Justice and Delinquency Prevention. *https://www.ojjdp.gov/ojstatbb/jrfcdb.

[CR61] Robson, D. A., Allen, M. S., & Howard, S. J. (2020). Self-regulation in childhood as a predictor of future outcomes: A meta-analytic review. *Psychological Bulletin,**146*(4), 324. 31904248 10.1037/bul0000227

[CR62] Satorra, A. (2000). Scaled and adjusted restricted tests in multi-sample analysis of moment structures. In *Innovations in multivariate statistical analysis: A Festschrift for Heinz Neudecker* (pp. 233–247). Springer.

[CR63] Seaton, E. K. (2020). A luta continua: Next steps for racism research among Black American youth. *Child Development Perspectives,**14*(4), 244–250. 10.1111/cdep.12388

[CR64] Smith-Bynum, M. A., Lambert, S. F., English, D., & Ialongo, N. S. (2014). Associations between trajectories of perceived racial discrimination and psychological symptoms among African American adolescents. *Development and Psychopathology,**26*(4pt1), 1049–1065. 24955844 10.1017/S0954579414000571 PMC4205197

[CR65] Stewart, E. K., Kotelnikova, Y., Olino, T. M., & Hayden, E. P. (2024). Early childhood impulsivity and parenting predict children’s development of externalizing psychopathology. *Development and Psychopathology,**36*(3), 1249–1261. 10.1017/S0954579423000482 10.1017/S0954579423000482 37144393

[CR66] Thompson, K., Roemer, A., & Leadbeater, B. (2015). Impulsive personality, parental monitoring, and alcohol outcomes from adolescence through young adulthood. *Journal of Adolescent Health,**57*(3), 320–326. 10.1016/j.jadohealth.2015.05.005 10.1016/j.jadohealth.2015.05.005 26143959

[CR67] Tiwari, B. B., McDowell, C., Roberts, O. S., Kogan, S., Chen, Z. A., & Rajbhandari-Thapa, J. (2024). A standard measure of structural racism, do we have one? Can we have one? A narrative review of commonly used measures and domains of use. *Journal of racial and ethnic health disparities*. 10.1007/s40615-024-02179-7 10.1007/s40615-024-02179-7 39285153

[CR68] Ursache, A., Barajas-Gonzalez, R. G., & Dawson-McClure, S. (2022). Neighborhood influences on the development of self-regulation among children of color living in historically disinvested neighborhoods: Moderators and mediating mechanisms. *Frontiers in Psychology,**13*, 953304. 10.3389/fpsyg.2022.953304 36389468 10.3389/fpsyg.2022.953304 PMC9643166

[CR69] Vines, A. I., Ward, J. B., Cordoba, E., & Black, K. Z. (2017). Perceived racial/ethnic discrimination and mental health: A review and future directions for social epidemiology. *Current Epidemiology Reports,**4*, 156–165. 28920011 10.1007/s40471-017-0106-z PMC5596659

[CR70] Weinberger, E. C. (2023). Developmental trajectories of conduct problems across racial/ethnic identity and neighborhood context: A systematic review. *Aggression and Violent Behavior,**71*, 101844. 10.1016/j.avb.2023.101844

[CR71] Williams, D. R. (2018). Stress and the mental health of populations of color: Advancing our understanding of race-related stressors. *Journal of Health and Social Behavior,**59*(4), 466–485. 10.1177/0022146518814251 30484715 10.1177/0022146518814251 PMC6532404

[CR72] Williams, D. R., & Mohammed, S. A. (2013). Racism and health I: Pathways and scientific evidence. *American Behavioral Scientist,**57*(8), 1152–1173. 10.1177/0002764213487340 10.1177/0002764213487340 PMC3863357 24347666

[CR73] Wills, T. A., Simons, J. S., Sussman, S., & Knight, R. (2016). Emotional self-control and dysregulation: A dual-process analysis of pathways to externalizing/internalizing symptomatology and positive well-being in younger adolescents. *Drug and Alcohol Dependence,**163*, S37–S45. 27306730 10.1016/j.drugalcdep.2015.08.039 PMC4911542

[CR74] Wills, T. A., Ainette, M. G., Mendoza, D., Gibbons, F. X., & Brody, G. H. (2007). Self-control, symptomatology, and substance use precursors: Test of a theoretical model in a community sample of 9-year-old children. *Psychology of Addictive Behaviors*, *21*(2), 205–215. wills@aecom.yu.edu. 10.1037/0893-164X.21.2.205. http://search.ebscohost.com/login.aspx?direct=true&db=psyh&AN=2007-08148-009&site=ehost-live 10.1037/0893-164X.21.2.205 17563140

[CR75] Wint, K. M., Opara, I., Gordon, R., & Brooms, D. R. (2022). Countering educational disparities among Black boys and Black adolescent boys from pre-k to high school: A life course-intersectional perspective. *The Urban Review*, 1–24. 10.1007/s11256-021-00616-z PMC8450170 34565917

