# Supplementary material for: Family-Centered Prevention Attenuates the Association Between Structural Racism Risk and Black Adolescents’ Low Self-regulation and Externalizing Behaviors: Secondary Analysis of a Randomized Clinical Trial
Source: Prev Sci. 2025 Jul 17;26(6):932–42. doi: 10.1007/s11121-025-01828-5 (PMC12394331; doi:10.1007/s11121-025-01828-5)
Supplement: Supplementary file 1 — Supplementary file1 (DOCX 113 KB) [file 11121_2025_1828_MOESM1_ESM.docx]

**Trial Protocol**


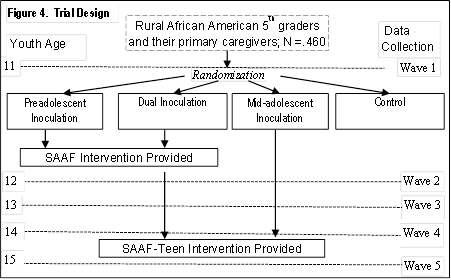
A random sample of 472 African American youth in 5^th^ grade and their primary caregivers were recruited for the study. Families were sampled from 8 Georgia counties. Figure 4 presents an overview of the study design. Youth and their primary caregivers were recruited in random order from lists of students in public schools in the targeted counties. Very few (<2%) rural African American youth in these areas attend private schools; thus, lists of public-school students approximate a population frame. Eligibility criteria for youth include age of 10-11 years and identification by the family and school as African American. Primary caregivers are not required to identify as African American. Siblings and stepsiblings who both are in the 5th grade were excluded.

**Random assignment.** After pretest, families were assigned randomly to one of four experimental groups: (a) *preadolescent inoculation*, (b) *mid-adolescent inoculation*, (c) *dual inoculation* control, and (d) control. Preadolescent inoculation families were assigned to receive SAAF at age 11 with no further intervention. Mid-adolescent inoculation families received SAAF–Teen at age 14. The dual-inoculation group were assigned to receive both SAAF at age 11 and SAAF–Teen at age 14. The control condition involved no intervention contact. Participants were notified if they were selected to participate in SAAF shortly after pretest. For each intervention implementation, families who were not attending a program received written information by mail regarding adolescent development and alcohol use. Sending these materials was intended to maintain investment in the project for families who were not attending an intervention. No alcohol use effects were expected based on receiving written materials. All families were followed across the course of the proposed study and assessed regardless of the number of intervention sessions they attend (intent-to-treat design).

**Repeated assessments and attrition.*
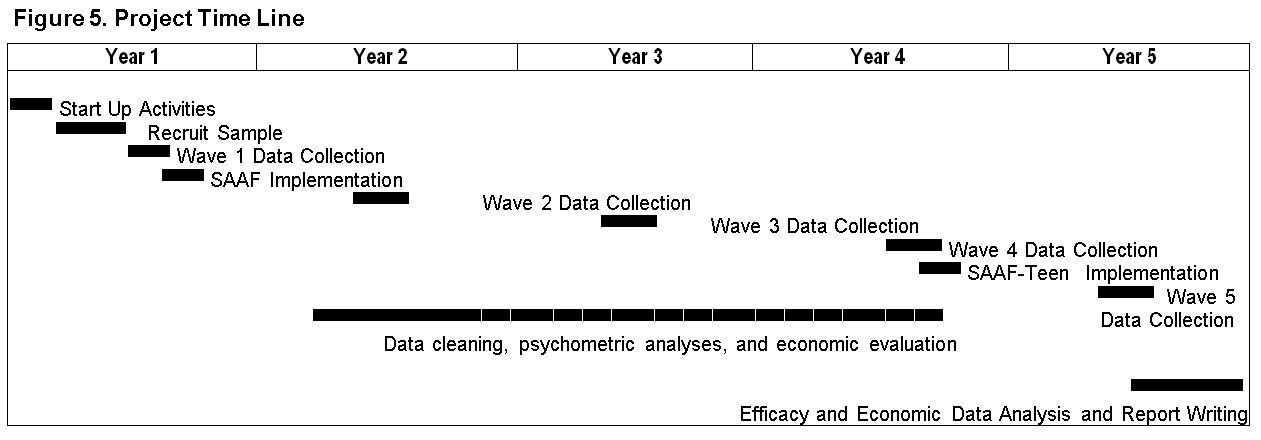
*** Families participated in 5 annual assessments. Wave 1, the baseline assessment occurred when youth were age 11 and approximately 3 months prior to implementation of SAAF. Wave 4 occured just prior to the implementation of SAAF–Teen. The final assessment, Wave 5, occured approximately 10 months after implementation of SAAF—Teen.

**Family Recruitment and Retention.** The recruitment procedures were similar to protocols used in our previous prevention trials with rural African American families. These protocols rely on community liaisons (CLs), residents of the counties from which families are sampled; they serve as contacts between CFR and the communities. They are selected on the basis of their positive reputations and extensive social contacts in their communities. The CLs work with the recruitment coordinator to enroll families, then remain in contact with participating families and track changes in the addresses and phone numbers of families who move. Family recruitment was conducted as follows:

1. Sampling lists. Research staff worked with school staff to identify eligible youth and develop a list from which to sample. We have excellent relationships with schools in the sampled counties.
2. Introductory letter. Caregivers of African American youth receive da letter introducing CFR, emphasizing its focus on health and resilience in African American communities, and informing caregivers that researchers are interested in screening families in the community for participation in a research project. Families were advised to expect a phone call from a local representative (the CL).
3. Phone screening. CLs followed up letters with phone screenings to primary caregivers to determine potential eligibility. CLs called families from participant lists in random order to ensure diverse representation of available families. If a family was potentially eligible, a screening/enrollment home visit is scheduled.
4. Home visit. At the home visits, research staff confirmed eligibility, described the project in detail, and answered any questions the family members may have had. Family members were informed that all project participants were expected to participate in annual data collection home visits over the next 5 years. In addition to these home visits, some families, selected by “chance,” would be asked to participate in a prevention program when their youth enter 6^th^ grade, a similar program 3 years later, or both programs. Families had to agree to attend if they were selected for either of the programs. If parents and youth agreed, a pretest assessment was implemented. Informed consent/assent was obtained at pretest. At each data collection, parents received $80 and target youth received $20.

**Retention of trial participants.** At each data collection, family members provide contact information for three or more individuals who will always know their whereabouts. We gathered email addresses, cell phone numbers, and Internet social networking addresses for participants and their primary contacts. Following each data collection visit, project staff contacted participants to inquire about the quality of the experience, confirm contact information, and answer questions. Between data collections, we phoned participants every 3 months to update contact information. Participants also received a project newsletter that includes a stamped address correction form, as well as birthday cards and holiday cards.

**Preventive Intervention Implementation Procedures.** Procedures for recruiting and training intervention facilitators, insuring curriculum fidelity, and engaging participants are based on our experiences in previous trials with rural African American families including the previous evaluation of SAAF and SAAF–T ^(1, 2, 3)^. SAAF/SAAF–T intervention facilitators are African Americans with a minimum education level of high school graduation. They are selected based on information from interviews and references that attest to their communication skills, engaging personalities, and ability to implement a structured program with fidelity. Facilitators received 30 hours of training on the implementation of each program. Training included curriculum activities, guided practice in delivering and pacing curriculum segments, and facilitator self-care. They worked from detailed manuals that describe all facets of program delivery. Didactic material, role-playing exercises, and modeling were used to teach the protocol for each session. To ensure ongoing quality control and fidelity, facilitators met with the Prevention Coordinator weekly.

**Intervention engagement.** Families who were randomized to an intervention condition received an introductory visit from their assigned facilitator prior to the first group session. This visit was designed to familiarize parents with the program, answer questions about the intervention, and increase the family’s comfort with the facilitator. During the visit, family members viewed a promotional video describing the program. They were asked about their weekly schedules, and sessions were planned to accommodate the schedules of as many families as possible. To facilitate attendance, during the first half hour of each intervention session, a meal was served to family members attending the session and any siblings of the target youth who accompany their parents. Assistance with transportation and on-site childcare was provided on request. Facilitators called participants before the program began and prior to each weekly session to encourage their involvement. Reminder postcards were sent from the CFR each week, highlighting the focus of the upcoming meeting. Facilitators called participants who missed a meeting to remind them of the next meeting and address any barriers to attendance. A $25 honorarium was provided to each family for each session attended, to defray any expenses associated with attendance.

**Prevention programs and program fidelity.** Standardized procedures insure program fidelity. The intervention manuals include a detailed format to be used for each session. Each facilitator was provided with materials designed to support correct execution of the session protocol, including an outline of each session, a checklist of the materials necessary for each activity, the specific theme of each task, and forms for in-session notes. Presentation of targeted processes on videotapes helps to ensure fidelity. Each session was audiotaped. Each team of facilitators was assigned an intervention supervisor, an expert trainer with a graduate degree in applied behavioral science, who reviewed session audiotapes with facilitators and provided weekly supervision of the intervention process. To assess program fidelity, session audiotapes were scored for protocol adherence using instruments validated in the SAAF and SAAF–Teen trials. Two judges dscore 20% of the sessions to assess interrater reliability (κ).

**Data Collection Procedures.** At each of the five assessments, one 2-hour home visit was made to each family for data collection. To minimize cultural bias, African American community members, many of whom have worked at CFR for years, served as field researchers. Field researchers received 16 hours of training and supervised practice that addressed obtaining informed consent, building rapport, and helping participants to use the computer-based survey system. Field researchers met weekly with the Project Coordinator to discuss any problems that arose. Data was gathered via audio computer-assisted self-interviewing (ACASI) technology. The user-friendly program guides respondents through the survey; those with low literacy skills are assisted through audio enhancements. *Consent/Assent.* Participating caregivers consented in writing to their own and their youths’ participation. Youth assented in writing to participation.

**Data Analysis.** Data was organized and cleaned following quality control procedures that involve analyses of outliers and unexpected or inappropriate missing-data patterns. Next, items’ distributions and constructs’ psychometric properties were examined. We established equivalence among experimental conditions with ANOVAs and chi-square tests on baseline levels of alcohol use and demographic variables. All primary analyses were intent-to-treat. Data were missing consistent with a “missing at random” (MAR) assumption (4), allowing us to handle missing data using full maximum likelihood (FIML) methods.**Aim 1.** Mean differences in alcohol use by experimental group at wave 5 (~10 months after SAAF–T implementation) were tested with structural equation models (SEM). For experimental comparisons among four groups, a sample size of 100-115 families per group is sufficient to detect an effect of .13 with .80 power (alpha < .05) and a power of .90 for an effect as small as .15. **Aim 2.** This aim examines pathways through which different inoculation strategies affect distal (W5) alcohol use. SEM provides a parsimonious tool that enables the analyst to identify indirect pathways of influence between intervention assignment and high school alcohol use outcomes. Significance of mediational effect tests are assessed with bootstrapping methods per MacKinnon ^(5)^. With Monte Carlo simulation ^(6)^ (*n* = 100 per group), we evaluated power to detect the significance of path coefficients that vary in their “true” magnitude, holding the other parameters in the model constant at values estimated from previous research ^(7, 8)^. For the paths included in Aim 2, power ranged from .7 to .9 for detecting path coefficients ranging from .2 to .4 (*p* < .05).

**References**

1. Mrazek PJ, Haggerty RJ. Reducing risks for mental disorders: Frontiers for preventive intervention research: National Academy Press; 1994.

2. Brody GH, Kogan SM, Chen Y-f, Murry VM. Long-term effects of the strong African American families program on youths' conduct problems. Journal of Adolescent Health. 2008;43(5):474-81.

3. Rohrbach LA, Grana R, Sussman S, Valente TW. Type II translation: transporting prevention interventions from research to real-world settings. Evaluation & the health professions. 2006;29(3):302-33.

4. Little RJ, Rubin DB. Statistical analysis with missing data: John Wiley & Sons; 2019.

5. MacKinnon DP, Lockwood CM, Hoffman JM, West SG, Sheets V. A comparison of methods to test mediation and other intervening variable effects. Psychological methods. 2002;7(1):83.

6. Jöreskog KG, Sörbom D. LISREL 8: User's reference guide: Scientific Software International; 1996.

7. Brody GH, Murry VM, Kogan SM, Gerrard M, Gibbons FX, Molgaard V, et al. The Strong African American Families Program: a cluster-randomized prevention trial of long-term effects and a mediational model. Journal of consulting and clinical psychology. 2006;74(2):356.

8. Beach SR, Hurt TR, Fincham FD, Franklin KJ, McNair LM, Stanley SM. Enhancing marital enrichment through spirituality: Efficacy data for prayer focused relationship enhancement. Psychology of Religion and Spirituality. 2011;3(3):201.
