# Supplementary material for: Family-Centered Prevention Attenuates the Association Between Structural Racism Risk and Black Adolescents’ Low Self-regulation and Externalizing Behaviors: Secondary Analysis of a Randomized Clinical Trial
Source: Prev Sci. 2025 Jul 17;26(6):932–42. doi: 10.1007/s11121-025-01828-5 (PMC12394331; doi:10.1007/s11121-025-01828-5)
Supplement: Supplementary file 2 — Supplementary file2 (DOCX 31 KB) [file 11121_2025_1828_MOESM2_ESM.docx]

**Supplement S2.**

Flow diagram of the Strong African American Families Programs randomized trial.

Assessed for Eligibility

(N = 825)

Randomized

Control

(N =220)

SAAF

(N= 252)

Unable to Contact

(N=25)

W2, (n=; unable to contact)

Excluded (N=353)

Ineligible (N=200)

Refused to Participate (N=153)

472 Families Enrolled and Pretested

Unable to Contact

(N=14)

Time 3 Follow-up, N=227

Time 3 Follow- up, N=206
